# Genomic epidemiology of *Streptococcus agalactiae* ST283 in Southeast Asia

**DOI:** 10.1038/s41598-022-08097-0

**Published:** 2022-03-09

**Authors:** Pakorn Aiewsakun, Wuthiwat Ruangchai, Yuttapong Thawornwattana, Bharkbhoom Jaemsai, Surakameth Mahasirimongkol, Anchalee Homkaew, Paveesuda Suksomchit, Padungsri Dubbs, Prasit Palittapongarnpim

**Affiliations:** 1 Department of Microbiology, Faculty of Science, Mahidol University, 272, Rama VI Road, Ratchathewi, Bangkok, 10400 Thailand; 2 Pornchai Matangkasombut Center for Microbial Genomics, Department of Microbiology, Faculty of Science, Mahidol University, 272, Rama VI Road, Ratchathewi, Bangkok, 10400 Thailand; 3 Department of Medical Sciences, Ministry of Public Health, 88/7, Tiwanon Road, Amphoe Muang, Nonthaburi, 11000 Thailand; 4 Microbiological Unit, Central Laboratory and Blood Bank, Faculty of Medicine, Vajira Hospital, Navamindraraj University, Bangkok, Thailand; 5 National Science and Technology Development Agency, Pathumthani, Thailand

**Keywords:** Microbiology, Infectious diseases, Molecular evolution, Phylogenetics

## Abstract

*Streptococcus agalactiae*, also known as Lancefield Group B *Streptococcus* (GBS), is typically regarded as a neonatal pathogen; however, several studies have shown that the bacteria are capable of causing invasive diseases in non-pregnant adults as well. The majority of documented cases were from Southeast Asian countries, and the most common genotype found was ST283, which is also known to be able to infect fish. This study sequenced 12 GBS ST283 samples collected from adult patients in Thailand. Together with publicly available sequences, we performed temporo-spatial analysis and estimated population dynamics of the bacteria. Putative drug resistance genes were also identified and characterized, and the drug resistance phenotypes were validated experimentally. The results, together with historical records, draw a detailed picture of the past transmission history of GBS ST283 in Southeast Asia.

## Introduction

Newborn infection by *Streptococcus agalactiae*, or Lancefield Group B *Streptococcus* (GBS), has been recognized as a significant health problem in the US and Europe since the mid-twentieth century^1,2^. The disease in newborns, characterized by pneumonia, septicemia, and meningitis, is likely caused by intrapartum or postpartum infection by bacteria that originally colonized the gut of pregnant women^3^. Nonetheless, recent studies have shown that the bacteria can cause diseases such as sepsis, arthritis, and meningitis in non-pregnant adults as well^4–10^.

There are five major clonal complexes (CCs) of GBS, namely CC1, CC10, CC17, CC19 and CC23, circulating in humans around the globe^11^. The sixth CC, CC26, is found to be circulating mainly in Africa^12^. It has been reported that the predominant and expanded clones within each of these CCs usually contain tetracycline resistance genes, such as *tet*(O) and *tet*(L), but most commonly *tet*(M)*,* typically carried by an integrative and conjugative element (ICE), mostly of the families Tn*916* or Tn*5801*^13^. Notably, these expanded clones form monophyletic clades including isolates from many countries around the world, and each clone appeared to share an ICE with a tetracycline resistance gene at the same genomic position^13^. This observation had led to a suggestion that acquisition of ICEs conferring tetracycline resistance facilitated parallel expansions of many human pathogenic GBS populations around the globe, selected and fixed through the extensive use of tetracycline in the mid-twentieth century^13^.

GBS has been recognized as a pathogen of non-pregnant adults since the early 2000s^9,10,14^. Compared to other genotypes, sequence type 283 (ST283), belonging to CC10^15,16^, is one of the most common strains causing invasive diseases in adults in Southeast Asia and Hong Kong, with the first patient dated back to 1995^17,18^. GBS is also an important veterinary pathogen. Several strains have been identified to cause mastitis in cow and streptococcosis in fish^19^. GBS CC67, and CC23 are among common strains capable of infecting cows^20^. Likewise, several strains of GBS have been identified in fish, including the human pathogenic strain ST7 serotype Ia, and non-haemolytic ST260 and ST261 serotype Ib^21^. In fact, GBS ST283 has also been isolated from diseased Tilapia (*Oreochromis* sp.)^21^, and is becoming increasingly recognized as an important fish pathogen, occasionally reported from fish farms without any outbreaks^22,23^. The fish and human isolates share the same mobile genetic elements and surface proteins, suggestive of an evolutionary linkage between the two^21^. Phylogenetic analyses suggested multiple transmissions of GBS ST283 between fish and humans^23^, but the precise nature of the transmission has not yet been determined. In 2015, there was a human outbreak of GBS ST283 in Singapore^16,24^. Epidemiological analyses suggested that the outbreak was significantly associated with consumption of raw freshwater fish, but not consumption of sashimi, sushi, or raw shellfish, or exposure to fish-related activities^16,24^.

In this study, we sequenced 12 samples of GBS ST283 capsular polysaccharide (CPS) type III isolated from adult patients in Thailand. Together with other publicly available data, we reconstructed a detailed transmission and evolutionary history of the bacteria in Southeast Asia.

## Results

### Genome sequencing

Complete genomes of 12 GBS isolates were sequenced in this study, all of which were obtained from non-pregnant Thai adult patients with sepsis, septic arthritis, or meningitis, that were treated in hospitals in Bangkok or nearby provinces in Thailand (Table 1). The patients were between 16 and 86 years old, (median age = 43.5 years); seven of them were female and five were male. All bacterial isolates were susceptible to penicillin G, amoxicillin, cefepime, cefotaxime, meropenem, chloramphenicol, clindamycin, erythromycin, linezolid, levofloxacin, and vancomycin. One isolate, A5, was found to be resistant to tetracycline. Sequencing summary statistics can be found in Table 1. All isolates belonged to CPS type III and were determined to be of the sequence type ST283 based on the allelic profiles of seven housekeeping genes, including *alcohol dehydrogenase gbs0054* (*adhP*), *phenylalanyl tRNA synthetase* (*pheS*), *amino acid transporter gbs0538* (*atr*), *glutamine synthetase* (*glnA*), *serine dehydratase gbs2105* (*sdhA*), *glucose kinase gbs0518* (*glcK*), and *transketolase gbs2105* (*tkt*).

**Table 1:** Whole genome sequencing summary statistics.

Sequence name/SRA accession number	Isolation date	Clinical metadata				Before cleaning						After cleaning					
		Sex	Age	Isolation source	Tetracycline Susceptibility	Forward reads			Reverse reads			Forward reads			Reverse reads		
						Read number	Read length (bp)	% duplicate reads	Read number	Read length (bp)	% duplicate reads	Read number (% remaining)	Read length (bp)	% duplicate reads	Read number (% remaining)	Read length (bp)	% duplicate reads
B105/SRR17330953	24/03/2013	M	69	Joint fluid	Susceptible	18,350,023	100	29.30	18,350,023	100	32.69	10,167,834 (55.41%)	70–100	36.22	10,167,834 (55.41%)	70–100	36.72
D23/SRR17330952	13/05/2014	F	86	Blood	Susceptible	18,303,560	100	28.39	18,303,560	100	29.94	11,345,952 (61.99%)	70–100	33.71	11,345,952 (61.99%)	70–100	34.13
E4/SRR17330949	30/05/2016	M	60	Blood	Susceptible	23,678,556	100	33.14	23,678,556	100	32.47	16,032,920 (67.71%)	70–100	34.69	16,032,920 (67.71%)	70–100	34.34
E5/SRR17330948	30/05/2016	F	69	CSF	Susceptible	26,829,615	100	23.88	26,829,615	100	24.23	17,701,877 (65.98%)	70–100	23.90	17,701,877 (65.98%)	70–100	23.82
B117/SRR17330947	21/05/2013	M	36	Blood	Susceptible	400,706	75–151	79.94	400,706	75–151	80.73	115,293 (28.77%)	70–151	91.49	115,293 (28.77%)	70–151	91.77
B140/SRR17330946	07/06/2013	F	48	Blood	Susceptible	2,065,945	75–151	52.42	2,065,945	75–151	53.80	705,121 (34.13%)	70–151	73.26	705,121 (34.13%)	70–151	72.97
A5/SRR17330945	23/10/2012	F	16	Blood	Resistant	1,362,953	75–151	57.82	1,362,953	75–151	59.36	462,165 (33.91%)	70–151	76.72	462,165 (33.91%)	70–151	76.69
A26/SRR17330944	29/10/2012	F	32	Blood	Susceptible	1,113,101	75–151	64.61	1,113,101	75–151	65.98	339,938 (30.54%)	70–151	83.20	339,938 (30.54%)	70–151	83.38
C62/SRR17330943	08/12/2013	M	24	CSF	Susceptible	1,730,536	75–151	56.06	1,730,536	75–151	58.03	565,390 (32.67%)	70–151	76.62	565,390 (32.67%)	70–151	76.84
D44/SRR17330942	02/07/2014	M	47	CSF	Susceptible	634,553	75–151	73.96	634,553	75–151	75.11	193,716 (30.53%)	70–151	88.62	193,716 (30.53%)	70–151	88.85
E19/SRR17330951	20/09/2016	M	23	CSF	Susceptible	1,920,382	75–151	53.07	1,920,382	75–151	55.36	616,774 (32.12%)	70–151	74.72	616,774 (32.12%)	70–151	74.95
PK/SRR17330950	08/08/2018	M	28	Joint fluid	Susceptible	17,285,460	100	25.58	17,285,460	100	25.36	9,097,011 (52.63%)	70–100	39.75	9,097,011 (52.63%)	70–100	41.87

Low quality reads in the datasets were removed by using Trimmomatic^40^ with the following parameters: PE = phred33; SLIDINGWINDOW = 4:30; MINLEN = 70.

### Phylogeny of GBS ST283 circulating in Southeast Asia

**Figure 1:**
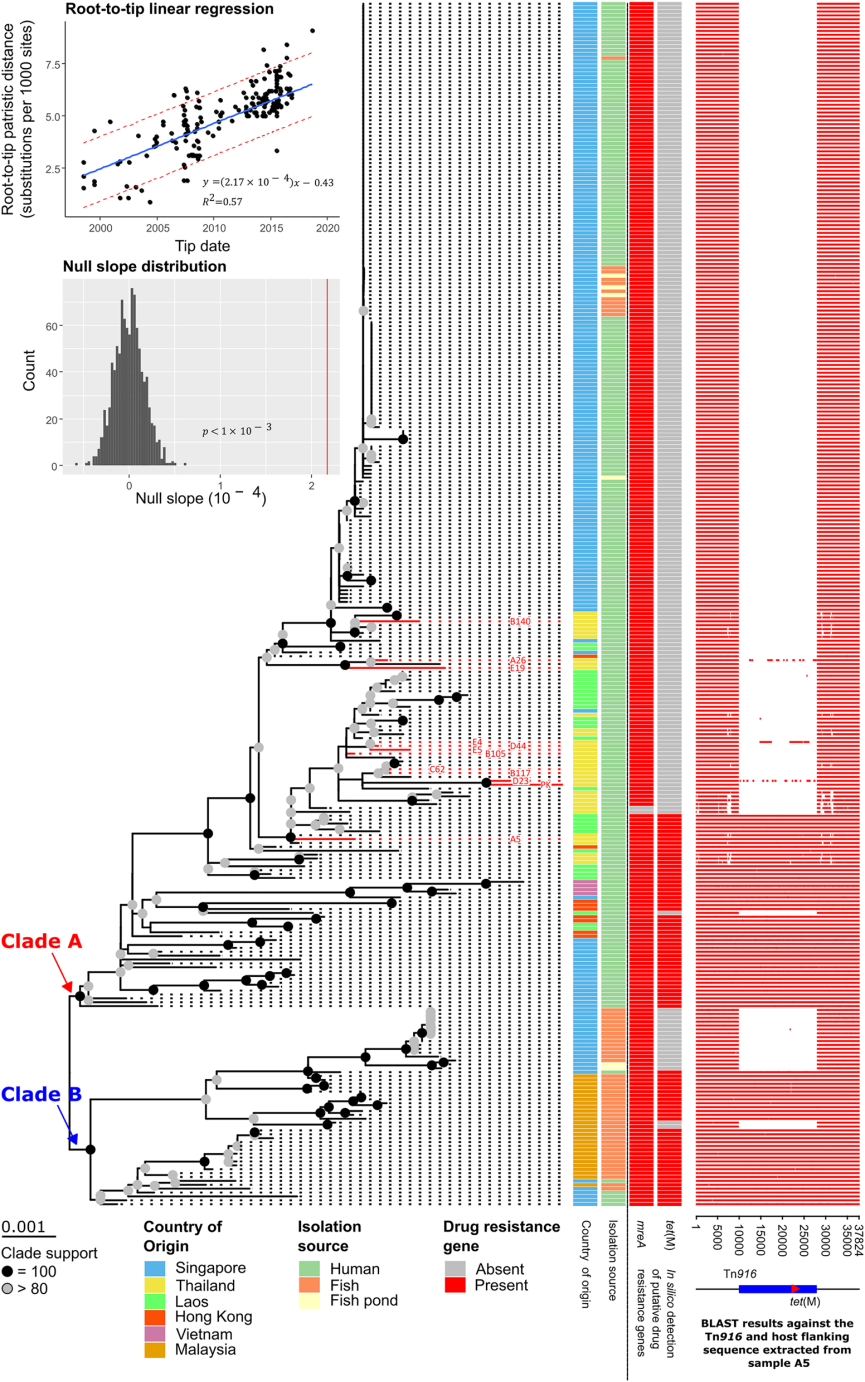
Phylogeny of GBS ST283 circulating in Southeast Asian countries. The tree (left) contains 310 isolates of GBS ST283**. **12 of them were generated by this study (red branches and red sequence names), all of which were obtained from Thai adult patients. The branches leading to two major phylogenetically distinct clades of GBS ST283 (A and B) are labelled. The tree was reconstructed under the maximum likelihood framework implemented in IQ-TREE2^45^ with the TIMe + ASC + R3 nucleotide substitution model, the best-fit model as determined under the Bayesian information criterion by ModelFinder^46^. The tree was rooted by maximizing the temporal signal in the dataset (root-to-tip regression analysis by minimizing residue mean square errors: slope = 2.17 × 10^–4^; R^2^ = 0.57; p < 10^–3^, inset graphs). The scale bar is in units of substitutions per site. Bootstrap clade support values were computed based on 1000 bootstrapped trees using the ultrafast bootstrap method, implemented in IQ-TREE2^45^. Nodes with 100% bootstrap support are labelled with black solid circles, while those with > 80% are labelled with grey circles. The country of origin, isolation source, and putative drug resistance genes potentially present in their genomes, namely *mreA* and *tet*(M), are shown (four columns in the middle of the figure; see keys for details). BLASTn analysis (right) showed that *tet*(M) is embedded within the ICE Tn*916* and that the absence of the gene corresponds precisely with the absence of the entire transposon. Sequence from A5 (Fig. 3) was used as a query in the BLASTn search. The figure was generated using R with the *ggplot2* 3.3.5^54^, and *ggtree* 2.4.1^55^ R libraries, and was arranged and labeled in Inkscape 1.0.2-2^56^.

We retrieved 298 whole genomes of GBS ST283 reported by Barkham et al. (2019) from the NCBI database and, together with the 12 datasets generated by this study, we reconstructed a phylogeny of GBS ST283 circulating in Southeast Asia (Fig. 1). The tree contains 310 bacterial isolates (Supplementary Table S1), sampled from 6 different countries/self-governing territories, including Hong Kong (9 sequences), Laos (30 sequences), Malaysia (28 sequences), Singapore (202 sequences), Thailand (37 sequences), and Vietnam (4 sequences). In terms of isolation source, 250 were from human; 54 were from fish, including carp, tilapia, and snakehead; and 6 were fish pond water samples.

The phylogeny was estimated based on a manually curated multiple sequence alignment of single nucleotide variants (SNVs) with potential recombination regions and long stretches of predominantly-gap positions removed (Supplementary Data S1). The genome of GBS ST283 SG_M1 (GenBank accession number: CP012419.2) was used as the reference genome in the SNV calling. Genome mapping coverages were between 94.29–100% (median = 99.93%). Average genome-wide read mapping depths were between 8.69 and 1573.02 (median = 135.95). Excluding unmapped regions, average read mapping depths were between 9.13 and 1589.17 (median = 136.92). Genome mapping summary statistics can be found in Supplementary Table S2. The tree was rooted by maximizing the temporal signal in the dataset (root-to-tip regression analysis by minimizing residue mean square errors: slope = 2.17 × 10^–4^; R^2^ = 0.57; p < 10^–3^, Fig. 1). The estimated tree topology is very similar to the one previously reported by Barkham et al.^15^. Analysis showed that all of the Thai isolates reported in this study (Fig. 1, red branches) clustered with other Thai isolates.

**Figure 2:**
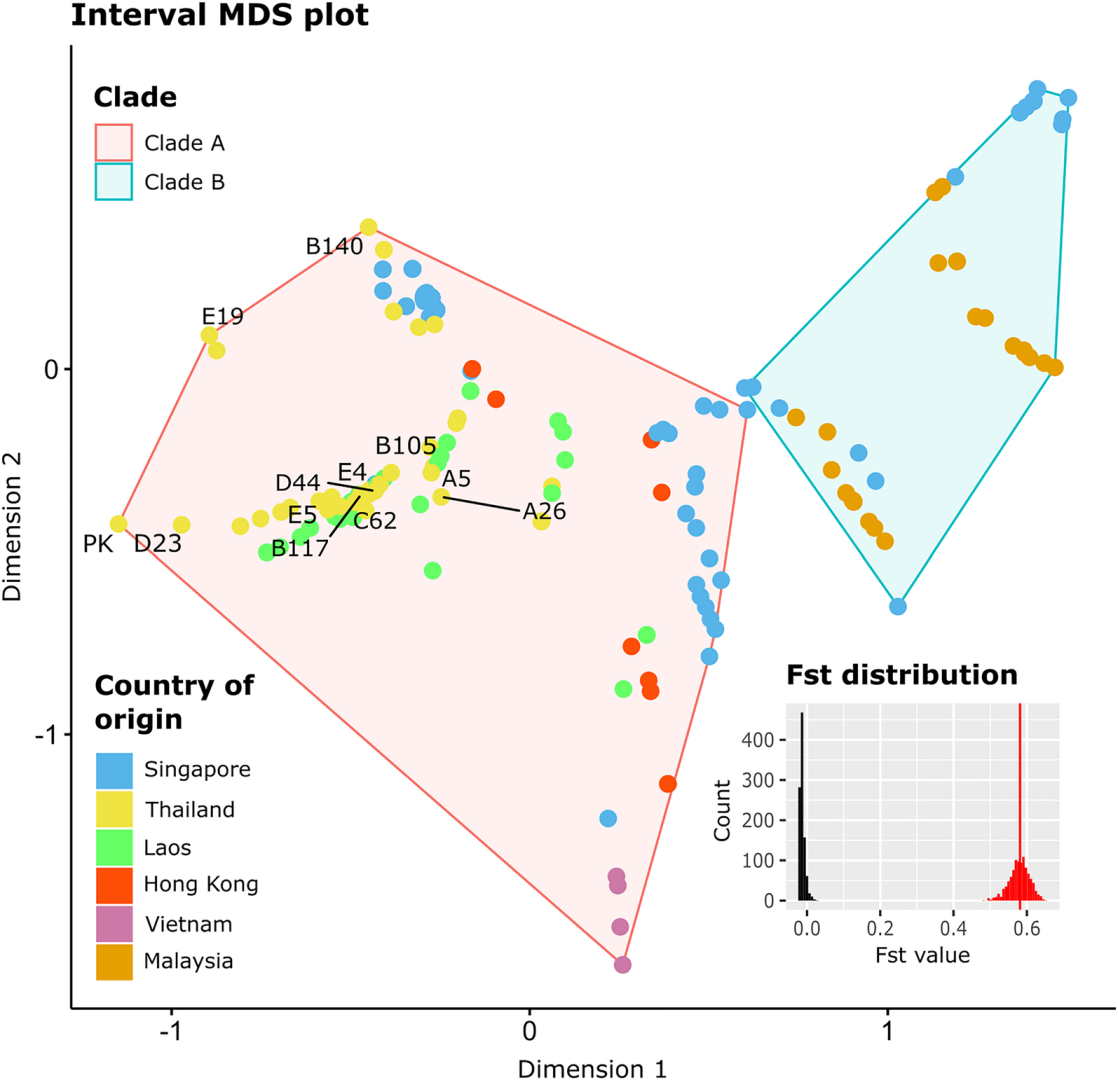
Interval multidimensional scaling plot and fixation index. The function *mds* in the *R* library *smacof* was used to perform interval multidimensional scaling (MDS) on the bacterial pairwise genetic distances extracted from the GBS ST283 tree (Fig. 1). The colors of the data points correspond to the bacterial country of origin (see key). The two convex hulls indicate the two major clades of GBS ST283 as shown in Fig. 1 (red: clade A, blue: clade B). The names of the 12 bacterial isolates whose genomes were sequenced in this study are shown. Pairwise fixation index (Fst) between the two clades was computed by using the function *stamppFst* implemented in the R library *StAMPP* (Fst = 0.58, inset histogram, red vertical line). The confidence interval of the Fst estimate was computed by using 1000 bootstrapped genetic datasets (95% confidence interval = 0.52–0.63, red histogram). Randomization test suggested that the clade assignment explained a significant portion of the bacterial genetic diversity (N = 1000, range null Fst values = − 0.022–0.026, p-value < 0.001, black histogram). The figure was generated using R with the *ggplot2* 3.3.5^54^ R library, and was arranged and labeled in Inkscape 1.0.2-2^56^.

The estimated phylogeny showed two distinct clades of GBS ST283. One clade (100% bootstrap support), tentatively designated as Clade A (Fig. 1, and Supplementary Table S1), contains 259 isolates, 244 of which were human isolates from Hong Kong (9 sequences), Laos (30 sequences), Singapore (179 sequences), Thailand (37 sequences), and Vietnam (4 sequences). Only a small number of bacteria in this clade were fish (11 carp isolates) and fish pond (4 isolates) isolates, relating to a large outbreak in Singapore. In contrast, the other clade (100% bootstrap support), designated as Clade B (Fig. 1, and Supplementary Table S1), contained proportionally more fish related samples. Clade B contained 28 fish isolates from Malaysia (all tilapias), and 23 isolates from Singapore; 15 of which were fish isolates (5 carps, 4 snakeheads, and 6 tilapias), 2 were fish pond isolates, and only 6 were human isolates. A multidimensional scaling plot depicting the genetic diversity of the two clades is shown in Fig. 2. Their pairwise fixation index was estimated to be 0.58 (95% confidence interval = 0.52–0.63, Fig. 2), significantly different from those computed under the random clade assignment (N = 1000, range of null Fst values = − 0.022–0.026, p-value < 0.001), meaning that the clade assignment explained a significant portion of the bacterial genetic diversity.

### In silico detection of putative drug resistance genes

ResFinder^25^ was used to screen for putative drug resistance genes in the bacterial genomes by mapping raw sequencing reads against a curated drug resistance gene sequence database of the program. To be considered as positive, contiguous full-length gene sequences must also be present in the genome assemblies and could be detected by BLASTn. The results are summarized in Supplementary Table S3.

Analysis showed that almost all of the samples investigated (308/310 genomes), including all of our samples, had the putative macrolide resistance gene *mreA*^26^ (Fig. 1). Drug susceptibility testing, however, showed that none of our isolates were resistant to macrolide antibiotics, including erythromycin, clindamycin, and linezolid, and thus were not analyzed further.

Another widespread drug resistance gene detected was *tet*(M) (Fig. 1), conferring resistance to tetracycline^27,28^. The gene was detected in 82/310 (26%) isolates; 27 of which were tilapia isolates (27/54 = 50% of all fish isolates) and 55 were human isolates (55/250 = 22% of all human isolates analyzed in this study). The bacteria identified as having *tet*(M) generally appeared to be phylogenetically basal to those without the gene, and this pattern could be observed in both of the two major clades of GBS ST283. This suggested that there were multiple independent events of losses of the *tet*(M) gene in the evolution of the bacteria. One of our samples detected as harboring *tet*(M) was indeed found to be resistant to tetracycline (A5, Table 1), while the others that were identified as lacking the gene were susceptible to the antibiotic, confirming the function of the gene.

### GBS ST283’s *tet*(M) is carried by Tn*916*

Many strains of GBS have been documented to possess *tet*(M), and the gene is mostly carried by two related ICEs belong to the families Tn*916* and Tn*5801*^13^. To examine the genomic context of *tet*(M) in GBS ST283, we assembled the bacterial genomes, and found that the assemblies of 81 out of 82 isolates detected as positive for *tet*(M) by ResFinder^25^ (Supplementary Table S3) were also identified by BLASTn as containing contiguous sequences (> 500 bases) showing high similarities to Tn*916* from *Enterococcus faecalis* strain DS16, which also carries *tet*(M) (GenBank accession number: U09422.1). The sequences exhibited more than 99.7% nucleotide identities, and covered more than 98.8% of the entire element length (18,032 nucleotides) with three contigs or fewer (Supplementary Table S4). The only exception was the assembly of SG-M1011 (SRA number: SRR8052446^15^), which was highly fragmented, and required numerous contigs with lengths of < 500 bases to cover the entire length of the reference Tn*916* element. Conversely, the *integrase* gene of Tn*5801.sag*, a member of the family Tn*5801* from *Streptococcus agalactiae* strain 14,774^29^ (GenBank accession number: HF930766.1), could not be detected in these assemblies by BLASTn. These results were consistent with that the *tet*(M) gene in GBS ST283 is carried by Tn*916* and not Tn*5801*.

**Figure 3:**
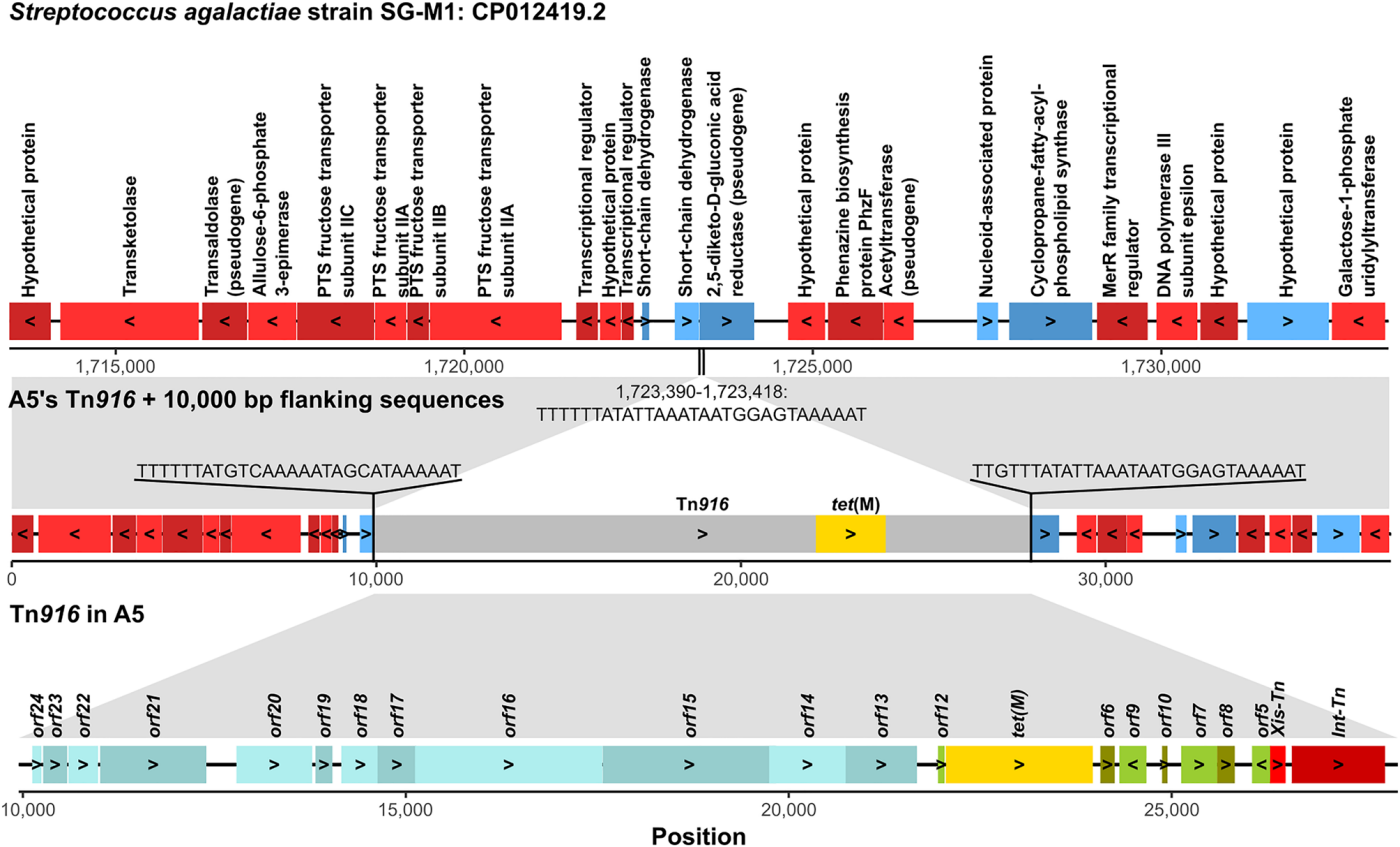
Tn*196* in GBS ST283. (Top) Schematic diagram illustrating an empty integration site in the reference GBS ST283 genome, strain SG-M1 (GenBank accession number: CP012419.2). Boxes represent protein coding regions, and the names of the protein products are shown on top. Genes’ directions are indicated by boxes’ colors and arrow heads drawn on the boxes (blue or “>”: forward orientation; red or “<”: reverse orientation). Sequence at the empty integration site (1,723,390 to 1,723,418) is shown. (Middle) Homologous position in A5, harboring Tn*916*. Homology between the reference genome and A5 is indicated by grey shadings. Protein coding regions in A5 were annotated by comparing its sequence to that of SG-M1 by BLASTn, showing > 99.9% nucleotide identity overall. Tn*916* is drawn in grey, and the *tet*(M) gene carried by the transposon is drawn in yellow. Direct repeats flanking the Tn*916* element are shown, exhibiting similarity detectable by BLASTn to the sequence at the empty integration site of SG-M1. (Bottom) Schematic diagram illustrating the genomic structure of Tn*916* in A5. Protein coding regions were annotated by comparing its sequence to the reference Tn*916* of *Enterococcus faecalis* DS16 (GenBank accession number: U09422.1) by BLASTn, showing > 99.9% nucleotide identity overall with 100% sequence coverage. The directions of the genes are indicated by arrow heads (“>”: forward orientation; “<”: reverse orientation) and the names of the genes are shown on top. Colors indicate to which functional modules the genes belong (blue: conjugation; green: transcriptional regulation; yellow: tetracycline resistance gene; grey: insertion and excision). The figure was generated using the *ggplot2* 3.3.5^54^ library in R, and was arranged and labeled in Inkscape 1.0.2-2^56^.

To further investigate the integration sites of these ICEs, the full-length sequence of Tn*916* was retrieved from the genome assembly of A5 with its flanking sequences (Fig. 3), and subsequently was used as a query in BLASTn searches against all other assembled genomes. Remarkably, the results showed that all of the Tn*916* elements found were located at the same genomic position (Fig. 1), mapping to the nucleotide position 1,723,390–1,723,418 in the reference GBS ST283 genome strain SG_M1 (CP012419.2), which does not have an ICE at this location (Fig. 3). For those that were identified as lacking the *tet*(M) gene by ResFinder^25^, BLAST analyses did not detect Tn*916* in the assemblies, yielding clean empty integration sites at the location (Fig. 1). Traces of contiguous sequences of > 500 nucleotides similar to Tn*916* were detected in D23, E4, and A26 (Supplementary Table S4). The sequences found, however, were highly fragmented, and did not cover the entire length of the Tn*916* element (Fig. 1). Coupled with the fact that none of the three isolates were found to resist tetracycline (Table 1), the presence of these sequences in their assemblies were likely due to contamination.

### Genomic epidemiology of GBS ST283 in human in Southeast Asia

**Figure 4:**
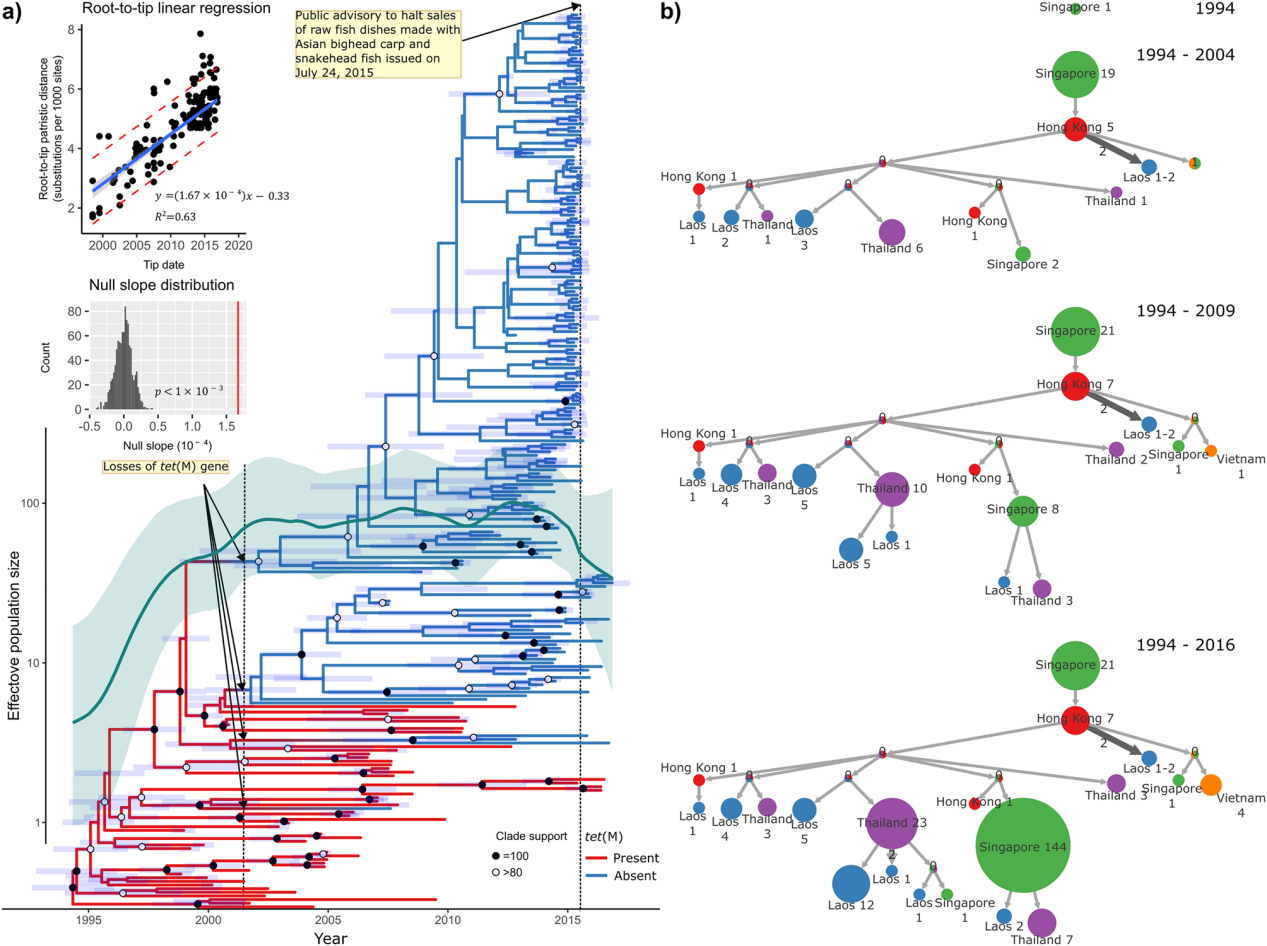
Genomic epidemiology of human GBS ST283 in Southeast Asians. (**a**) Time-calibrated phylogeny of 246 GBS ST283 infecting Southeast Asians. Significant temporal signal was detected by root-to-tip regression analysis (inset graphs: slope = 1.67 × 10^–4^; R^2^ = 0.63; p < 10^–3^). The phylogeny was inferred under the Bayesian phylogenetic framework by using BEAST 1.10^49^ with the TVMe substitution model, the Bayesian time-aware GMRF Skyride coalescent tree prior, and the random local relaxed clock model. Nodes with 100% clade support are labelled with black solid circles, while those with > 80% clade support are labelled with open circles. Branch lengths are in years. Node bars represent 95% highest posterior densities of the estimated dates. Branch colors indicate the inferred ancestral states of the *tet*(M) gene (see key). An approximate timepoint when the multiple losses of the *tet*(M) gene occurred is indicated. The timepoint when the public advisory to halt sales of dishes made with raw freshwater fish was issued in Singapore is also indicated. The inferred past population dynamics is also shown (thick green line = median effective population size; light green region = 95% highest posterior density). The figure was generated using R with the *ggplot2* 3.3.5^54^, and *ggtree* 2.4.1^55^ R libraries, and was arranged and labeled in Inkscape 1.0.2-2^56^. (**b**) Reconstruction of GBS ST283 epidemic locations over various timeframes. Four timeframes are shown as indicated on the right-hand side. The trees were pruned such that nodes whose branch starts outside the date range are excluded. The inferred histories are shown using compressed trees. Different colors correspond to different countries. Node size is approximately proportional to the number of samples contained in the node. Numbers on branches are the numbers of times their subtrees are found in the full tree. The tree figures were generated by PastML 1.9.33^53^ and were arranged and labeled in Inkscape 1.0.2-2^56^. Ancestral states of both the presence and absence of the *tet*(M) gene and the past epidemic locations were inferred under the maximum likelihood framework using PastML 1.9.33^53^ with the MPPA + F81 option, conditioning on the states of the collected samples and the time-calibrated tree shown in (**a**).

The epidemiological history of human GBS ST283 circulating in Southeast Asians was inferred, including the timing of the bacterial origin, its past population dynamics, and past geographical transmission history (Fig. 4). All fish and fish pond isolates, and the three human isolates (SRR8052386, SRR8052389, and SRR8052451) clustering with fish isolates, were removed from the analysis. This was because the model we used to describe the population dynamics of the bacteria (i.e. the Bayesian time-aware GMRF Skyride coalescent tree prior, see “Materials and methods” assumed that all individuals in the analysis belong to the same single population; mixing human and fish as well as fish-related samples in the analysis may violate such an assumption and yield an inaccurate description of the dynamics. The human isolate, PK, was also excluded, as it was the only isolate serendipitously collected in 2018. A root-to-tip regression analysis was reperformed and showed that there was still a significant temporal signal in the dataset (slope = 1.67 × 10^–4^; R^2^= 0.63; P < 0.001; and N = 1000), allowing us to infer the past epidemiological history of the bacteria. The past presence/absence of the *tet*(M) gene were also reconstructed.

Our analyses estimated the rate of evolution of GBS ST283 to be 1.26 × 10^–3^ (95% HPD: 1.05 × 10^–3^–1.51 × 10^–3^) substitutions per SNP site per year. The time to most recent common ancestor of the bacteria in this dataset was estimated to be 1994.34 (95% HPD: 1992.51–1995.90). The results showed that, for the first 6 to 7 years of the epidemic, the bacterial effective population size increased rapidly, but then the expansion rate dropped significantly, and the population size remained relatively constant after the early 2000s. The drop in the population growth rate coincided with multiple losses of the *tet*(M) gene (Fig. 4), suggestive of a link between the two phenomena. We also found that the bacterial population started to decline at around 2015, and this matched with the public advisory to halt sales of dishes made with raw freshwater fish in Singapore (Fig. 4).

In terms of geographical location, our analyses suggested that the origin of the outbreak was most likely in Singapore. Within the period of the rapid expansion (late 1990s to early 2000s), the bacteria spread to multiple countries, first, from Singapore to Hong Kong, and then from Hong Kong to Thailand and Laos. The bacteria subsequently spread to Vietnam in the late 2000s. Multiple cross-country transmissions between Singapore, Thailand and Laos in 2010s were also inferred.

## Discussion

GBS ST283 has become increasingly recognized as an important pathogen in Southeast Asia^30^. All of the 12 bacterial isolates reported in this study were collected from Thai adult patients (Table 1), treated in the hospitals in Bangkok or nearby provinces in the central part of Thailand. We found that all of them cluster with other Thai isolates reported by Barkham et al.^15^, which interestingly, were collected from the Eastern (Sa Kaeo) and North Eastern (Nakhon Phanom) parts of Thailand. This suggested that the bacteria could readily spread across geographical regions, and is not confined to specific locations. Furthermore, our analysis revealed that the Thai isolates form several well-supported phylogenetically distinct groups, clustering with other isolates from Laos and Singapore. This pattern is consistent with multiple cross-country transmissions of the bacteria.

Our analysis estimated the bacterial origin to be in Singapore, dating back to 1994.34 (95% HPD: 1992.51–1995.90, Fig. 4A). This date estimate is slightly younger than one reported by Barkham et al.^15^, but is still comparable (1985, 95% HPD: 1980–1990). The bacteria were then inferred to spread to at least three countries within a period of 10 years between 1994 and 2004 (Fig. 4B), firstly to Hong Kong, matching well with the historical medical record of the first set of invasive GBS infections in non-pregnant patients in Hong Kong in 1995^18^, and subsequently to Thailand and Laos. By the late 2000s, the bacteria were found in Vietnam, and several more cross-country transmissions between Singapore, Thailand and Laos were inferred to occur in 2010s. While the reconstructed transmission history may be incomplete due to the limited dataset that we used, this result nonetheless strengthens the notion that the bacteria could readily spread across countries.

While the ultimate origin of human pathogenic GBS is still unknown, it has been observed that, for many expanded GBS clones, onset of their initial spread coincided with the acquisition of *tet*(M), a tetracycline resistance gene^13^. *tet*(M) encodes a ribosome protection protein, which catalyzes the GTP-dependent release of tetracyclines from the ribosome, conferring a tetracycline resistance phenotype^31^. The gene was detected in one (A5) out of the 12 isolates reported in this study, and it was the only isolate that exhibited resistance to tetracycline (Table 1), validating the gene function.

Across the entire dataset, our analyses detected *tet*(M) in 82 out of 310 GBS ST283 isolates (26%), carried by an ICE of the family Tn*916* (Fig. 3). Very remarkably, we found that all isolates shared the same Tn*916* integration site (**Fig. ****1**). Since the integration of an ICE occurs randomly, although more frequently at the genomic locations with long stretches of A’s and T’s^32^, this observation strongly supported that the insertion of Tn*916* likely occurred before the expansion of GBS ST283. Indeed, our ancestral state reconstruction of the presence/absence of *tet*(M) also yielded a consistent result, inferring the gene to be present in the most recent common ancestor of the bacteria (Fig. 4). Such pattern has been noted in many distinct major expanded clones of GBS^13^. Together, our results further support the notion that the acquisition of ICEs conferring tetracycline resistance is likely an important landmark event that led to the emergence, expansion, and eventually fixation of many tetracycline-resistant pathogenic GBS lineages in humans around the world, and this phenomenon was likely driven by the extensive use of tetracycline starting in the late 1940s, previously proposed by Da Cunha et al*.*^13^.

Nevertheless, while we found that early GBS ST283 isolates generally contained *tet*(M), many of the more recent isolates lacked the gene (Fig. 1). Our analysis suggested that human GBS ST283 independently lost the *tet*(M) gene at least four times within a very short period of time at around early 2000s (Fig. 4A). This pattern corroborated well with the earlier observation made by Barkham et al*.*^15^ that human GBS ST283 in Southeast Asia appeared to lose its ability to resist tetracycline over time, although no explicit link between the loss of tetracycline resistance and the *tet*(M) gene was made. Examination of genome assemblies revealed that those that lacked the gene had a clean empty integration site, and did not have Tn*916* anywhere else (Fig. 1). This perfect correlation supported that it is the integration and excision of the ICE carrying the *tet*(M) gene, and not the gain or loss of the gene or the gene function itself, that underlies the tetracycline resistance phenotype of GBS ST283.

Furthermore, our analysis revealed that the losses of *tet*(M) roughly coincided with the sudden halt in bacterial expansion, followed by stabilization of the bacterial population (Fig. 4). This further supported the probable roles of *tet*(M) in the bacterial expansion^13^. In any case, it is worth noting that tetracycline is actually not used to treat GBS infection in human; the current drugs of choice are β-lactam antibiotics including penicillin and cephalosporins, followed by clindamycin, which is a lincosamide antibiotic if the patient is allergic to penicillin and/or cephalosporins, and lastly vancomycin if the other drugs are not viable options^33^. In fact, the clinical use of tetracycline has become quite limited, and its clinical usefulness has been declining as many important bacteria have now become tetracycline-resistant^34,35^. This might in part explain the observed multiple losses of the *tet*(M) gene in GBS ST283, as there would not have been strong evolutionary pressure to maintain it, either directly or indirectly.

Nonetheless, it should be noted that, for non-ST283 strains, the prevalence of tetracycline resistance is still very high despite the fact that the drug is not used to treat human GBS infection and that the medical use of tetracycline has been declining in general. Da Cunha et al*.*^13^ highlighted very high and stable rates of tetracycline resistance of > 90% in several human GBS strains. Similarly, Barkham et al.^15^ noted that 88% of non-ST283 human GBS isolates collected in Singapore between 2001 and 2018 were tetracycline resistant. It was thus suggested that Tn*916* might impose only a limited fitness cost, and once inserted into the genome, the element is maintained despite decreases in antibiotic selection pressure. These high rate estimates are, however, in stark contrast to the ones observed in GBS ST283. Among our 12 human ST283 isolates collected between 2012 to 2018 in Thailand, only one isolate was found to be tetracycline resistant (1/12 = 8.3%, Table 1). Across the entire dataset analyzed herein, only 55/250 = 22% of human ST283 isolates were identified as carrying the *tet*(M) gene, and if we were to focus only on isolates collected after 2010, the rate would drop to just 11/234 = 4.7%. Since the samples analyzed in this study were not systematically collected, and were relatively small, the precise differential prevalence of tetracycline resistance in ST283 and non-ST283 GBS could not be made. However, at face value, this big difference in the rates is indicative of potential differences in the bacterial biology and/or host and environmental adaption. Additional analyses of systematically collected samples may provide further insights into the underlying factors that shape the differential distribution of the antibiotic resistance in ST283 and non-ST283 GBS (if any).

Apart from *tet*(M), *mreA* was another putative drug resistance gene that was found ubiquitously among the bacteria examined (Fig. 1). The gene was originally proposed to confer a macrolide resistance phenotype. It was first discovered in *Streptococcus agalactiae* COH31 γ/δ, a strain that is resistant to macrolides, and its function in conferring macrolide resistance was further validated by cloning the gene into *Escherichia coli*^26^. However, the gene was later found in most, if not all, GBS isolates that were susceptible to macrolides^36,37^, characterized to encodes a flavokinase^36^, and its relationship to the macrolide resistance phenotype could not be replicated in *Enterococcus faecalis*^36^. Similarly, despite the fact that *mreA* was found in all of our 12 samples (Fig. 1), none were resistant to macrolide antibiotics, including erythromycin, clindamycin, and linezolid, further supporting the idea that the presence of the gene is not sufficient to confer a macrolide resistance phenotype, and the gene probably has a metabolic function instead^36^.

GBS ST283 has also been increasingly recognized as an important fish pathogen. Analysis showed that there were two major clades of GBS ST283 (Figs. 1 and 2). One contained predominantly human isolates, along with a few fish and fish pond isolates that were linked to a large outbreak in Singapore in 2015 (Clade A, Fig. 1). Conversely, the other clade contained mostly fish-related isolates with a few human samples (Clade B, Fig. 1). Although we observed differential prevalence of the two clades in fish samples, the number of samples in the analysis was probably too small and the collection protocols were perhaps too unsystematic to make any conclusive statements regarding their (potentially different) host preferences however.

In terms of the distribution of the antibiotic resistance gene *tet*(M) in fish GBS ST283 isolates, analyses of this dataset estimated an overall prevalence of the gene to be 27/54 = 50%, substantially greater than the overall 22% rate of *tet*(M) in human GBS ST283. As previously noted by Barkham et al*.*^15^, tetracyclines are among the antibiotics frequently used to control fish streptococcosis in Southeast Asia^38^, and sometimes even at very high concentrations exceeding the maximum limits^39^, and this may in part explain the high prevalence of *tet*(M) in the fish GBS ST283. Interestingly, none of the 11 fish isolates in Clade A, associated with a large outbreak of GBS in Singapore in 2015, were detected positive for *tet*(M) (0/11 isolates = 0%) (Fig. 1). All of the 27 fish isolates detected positive for the gene were from Clade B (Fig. 1); 26 of which were Malaysian tilapia GBS ST283 isolates collected between 2007 and 2008 (26/28 = 92.9%). In contrast, similar to the pattern observed in Clade A, all but one of the Singaporean fish isolates in Clade B, which were collected in 2015, were identified as lacking the gene (1 tilapia isolate/15 carp, snakehead, and tilapia isolates = 6.6%). These observations suggested that the distribution of *tet*(M) in fish GBS ST283 may vary through space, and time, as well as host species, and further systematic analyses are required to better characterize the distribution of the gene.

Regarding the history of the bacterial cross-species transmission, although a precise history could not be reconstructed due to the limited dataset we analyzed, the fact that human and fish-related samples did not form separate monophyletic clades, but instead clustered with one another, supported that there must have been multiple cross-species transmissions between the two^23^. Close inspection of the phylogeny (Fig. 1) revealed that, for both clades, most of the basal samples were those of human origin, consistent with that human variants may have ultimately given rise to the fish variants (although might not be directly). Furthermore, the observation that the halt of sales of raw freshwater fish dishes in Singapore coincided with the decline in the bacterial population at around the year 2015 (Fig. 4) indicated that fish-to-human transmission might have had also occurred, and that fish-associated pathogens may retain the full potential to infect humans. The relationships between human and fish pathogens are highly complex and certainly warrant further study. The continuous expansion of fish farming in the region, together with the facts that the bacteria can readily spread across countries and potentially also across different host species causing invasive diseases, highlights the need for continual surveillance and monitoring of GBS ST283 in Southeast Asia to more effectively control the disease.

## Materials and methods

### GBS isolates

Twelve invasive GBS isolates were obtained from a collection of 200 clinical samples from patients attending hospitals in Bangkok and nearby provinces in 2012 to 2016 with one isolate obtained in 2018. All isolates were collected during the course of treatment and “The Ethics Committee of the Faculty of Medicine Vajira Hospital, Navamindradhiraj University (approval ID COA 145/2564), Bangkok, Thailand” waived the informed consent.

### Study protocol

The study was approved by the ethic committee of the Faculty of Medicine Vajira Hospital, Navamindradhiraj University (approval ID COA 145/2564). All experimental protocols were performed in accordance with the relevant guidelines and regulations laid by the ethic committee. Clinical isolates used in this study were fully anonymised.

### Drug susceptibility testing

Antimicrobial drug susceptibility testing was performed according to the manufacturer’s instruction using a BD Phoenix M50 system (BD Diagnostic Systems, Sparks, MD, USA). Briefly, a few colonies of each isolate were resuspended in the ID broth (BD Diagnostic Systems) to a concentration of 0.5 McFarland. The adjusted inoculum (25 µl) was then added to antimicrobial susceptibility test (AST) broth, containing methylene blue and resazurin (BD Diagnostic Systems). The suspension was then added to the BD Phoenix GP-PMIC 84 panels to test if the bacteria were susceptible to amoxicillin, cefepime, cefotaxime, chloramphenicol, clindamycin, erythromycin, levofloxacin, linezolid, meropenem, penicillin G, tetracycline, and vancomycin. Quality controls were performed according to the manufacturer’s recommendations using the following reference isolates; *Staphylococcus aureus* ATCC 25923, *Escherichia coli* ATCC 25922, and *Pseudomonas aeruginosa* ATCC 27853. All of the twelve bacterial isolates in this study were verified as GBS using the BD Phoenix M50 system.

### DNA extraction

GBS isolates were sub-cultured twice from the stock cultures on 5% blood agar plates. A single colony from each isolate was inoculated into 5 ml brain heart infusion (BHI, Becton and Dickinson, USA) broth. After an overnight incubation with shaking at 37 °C, cells were pelleted by centrifugation at 3226×*g* for 10 min. The pellet was then washed with 1 ml of TNE (10 mM Tris pH 8.0, 10 mM NaCl, 10 mM EDTA) buffer and gently resuspended with 400 μl of TNE buffer. After an 80 °C incubation for 20 min, 50 μl of lysozyme (80 mg/ml) was added to the resuspension, which was then incubated at 37 °C for 2 h with mixing every 15 min. After the addition of 75 μl of 10% sodium dodecyl sulfate (SDS), the bacterial lysate was mixed by inversion and incubated at 65 °C for 3 h with mixing every 30 min. After phenol–chloroform extraction, 10 μl of 20 mg/ml of RNase was added to the supernatant followed by further incubation at 37 °C for 30 min. After phenol–chloroform extraction, genomic DNA was ethanol precipitated. Finally, the DNA pellet was resuspended in 10 mM Tris buffer, pH 8, and stored at 4 °C. The concentration and purity (OD260/280) of the genomic DNA was determined by Nano drop (Denovix DS-11 FX + , Thermo Fisher Scientific).

### Whole genome sequencing

Agarose gel electrophoresis was performed to confirm the absence of RNA and DNA degradation. 30 μl (50 ng/µl: OD260/280 ~ 1.8) of genomic DNA was sent for whole genome sequencing. DNA libraries of isolates: B117, B140, A5, A26, C62, D44, and E19, were prepared using a Nextera XT DNA library preparation kit (Illumina, USA). DNA tagmentation, library amplification, library clean up, library normalization, and library pooling, were carried out according to the manufacturer’s instruction. PhiX control V3 was added to the library to a final concentration of 1% as an external control, and the libraries were sequenced using a 150 bp paired-end NextSeq 550 sequencer (Illumina, USA). Genomic DNA samples of PK, B105, D23, E4, and E5 were sent for normal DNA library construction and 100 bp paired-end sequencing at BGI Genomics (Shenzhen, China) using the BGISEQ-500 platform. DNA concentration, purity, and integrity were checked by the company, and the DNBSEQ sequencing technology was used to generate PE100 data with 10–30 × coverage.

### Sequence typing assignment

Sequence type was determined based on seven housekeeping genes, namely *alcohol dehydrogenase gbs0054* (*adhP*), *phenylalanyl tRNA synthetase* (*pheS*), *amino acid transporter gbs0538* (*atr*), *glutamine synthetase* (*glnA*), *serine dehydratase gbs2105* (*sdhA*), *glucose kinase gbs0518* (*glcK*), and *transketolase gbs2105* (*tkt*). Gene sequences were reconstructed by mapping short reads to the reference genome GBS ST283 SG_M1 (GenBank accession number: CP012419.2), and submitted to PubMLST. The results revealed that all of the 12 isolates sequenced in this study are of the ST283 genotype.

### Single nucleotide variant calling

Whole genome sequences of 298 GBS ST283 in FASTQ format reported by Barkham et al.^15^ were retrieved from the NCBI database. Together with the 12 datasets generated by this study, we had 310 datasets of whole genome sequences of GBS ST283 in total (Supplementary Table S1). Low quality reads were removed by using Trimmomatic^40^ with the following parameters: PE = phred33; SLIDINGWINDOW = 4:30; MINLEN = 70. After the cleaning, remaining high-quality reads were mapped to the reference genome (GBS ST283 SG_M1; GenBank accession number: CP012419.2) using bwa with the *mem* algorithm^41^, skipping seeds with more than 100 occurrences (− c 100), marking split hits as secondary mappings (− M), and only reporting mapped reads with a minimum score of 50 (− T 50). The presence of PCR and optical duplicates were marked by using *MarkDuplicates*, implemented in GATK^42^. Single nucleotide variants (SNVs) were called by using bcftools with the multiallelic and rare-variant calling algorithm^43^. A multiple sequence alignment of SNVs was made and filtered by using *VariantFiltration* and *SelectVariants* functions, implemented in GATK^42^, removing sites located in repetitive and endogenous phage integration regions. Summary statistics of the genome mapping can be found in Supplementary Table S2.

### Maximum likelihood phylogenetic reconstruction

Potential recombinant regions within the alignment were checked using RDP, GENECONV, Chimaera, MaxChi, BootScan, SiScan, and 3Seq, all were implemented in Recombination Detection Program 4 ^44^. Events detected by more than four programs were considered significant. The alignment (Supplementary Data S1) was 718 nucleotides long after the removal of recombination regions and long stretches of predominantly-gap positions. Potential recombination was checked again in the curated alignment, and no further recombination events were found.

IQ-TREE2^45^ was used to reconstruct the phylogeny based on the manually curated sequence alignment. The best-fit nucleotide model was determined to be TIMe + ASC + R3 under the Bayesian information criterion by ModelFinder^46^, and was used for the tree reconstruction. Clade bootstrap support values were computed based on 1000 bootstrapped trees using the ultrafast bootstrap method, implemented in IQ-TREE2^45^. The tree was rooted by maximizing the temporal signal in the dataset, quantified by root-to-tip regression analysis (see “Temporal signal detection” section). Two major clades of GBS ST283 were identified from the tree (see main text). The function *mds* in the R library *smacof* was used to perform interval multidimensional scaling on the bacterial pairwise genetic distances extracted from the tree.

### Fixation index

Pairwise fixation index (Fst) between the two major clades of GBS ST283 was computed using the function *stamppFst* implemented in the R library *StAMPP*. The calculation assumed haploid bacterial genomes. Examination revealed that, excluding gaps, almost all sites were biallelic sites (717/718 sites). Only one site had three alleles, with the aggregated minor allele frequency of only 2.58% (“A”: 3/310; “T”: 5/310; and “G”: 302/310). The two alleles were considered as a single non-major allele, and all sites were considered biallelic. Confidence interval of the Fst estimate was computed based on 1000 bootstrapped genetic datasets. Randomization test was used to assess if the clade assignment could explain a significant proportion of the bacterial genetic diversity.

### Drug resistance gene detection

Whole genome short-read sequencing data of individual bacterial isolates were mapped to a curated list of drug resistance gene sequences using ResFinder and its gene database^25^. The significant thresholds for gene detection were 95% gene mapping coverage, 95% gene-wide nucleotide identity, and 0.5 gene mapping depth normalized by whole-genome mapping depth (computed across mapped regions only). To ensure that the detected genes were not due to false non-specific short-read mapping, the genome sequences were assembled (see below) and the presence of the full-length genes were verified by using BLASTn under the default settings. Genes were considered positive only when they were detected by both methods. See Supplementary Table S3 for the result summary.

### Whole genome de novo assembly

Genome sequences were assembled by using a de novo genome assembler IDBA-UD^47^ under its default settings with 3 k-mer values, including 20, 40, and 60.

### Characterizing the genomic context of the *tet*(M) gene in GBS ST283

About 26% of the genomes examined were detected as positive for the tetracycline resistance gene *tet*(M)*,* known to be typically carried by two related transposons namely Tn*916* and Tn*5801* in GBS^13^, and the distribution of the gene was highly complex. To characterize the genomic context of the *tet*(M) gene detected in our dataset, draft assembled genomes were searched for contiguous sequences similar to the reference Tn*916* element from *Enterococcus faecalis* strain DS16 (GenBank accession number: U09422.1) and the *integrase* gene of Tn*5801.sag* from *Streptococcus agalactiae* strain 14774 (GenBank accession number: HF930766.1) by using BLASTn^48^ under the default settings. See Supplementary Table S4 for the results. Only BLAST hits with > 500 bases are reported.

To further examine the integration site of the detected transposons, the full-length sequence of the transposon carrying the *tet*(M) gene was retrieved from the genome assembly of A5 with its 3′ and 5′ flanking sequences (10,000 bases), and subsequently was used as a query in BLASTn searches^48^ against other assembled genomes and also the reference GBS ST283 genome strain SG_M1 (CP012419.2) under the default settings.

### Tip-dating and estimation of past population dynamics

Epidemiological history of GBS ST283 circulating in humans in Southeast Asia was inferred. All fish-related isolates, and human isolates clustering closely with fish isolates, were removed from the analysis. The “PK” human isolate was also excluded, as it was the only sample serendipitously collected in 2018. Root-to-tip regression analysis (see “Temporal signal detection” section) still detected a significant temporal signal in the data.

A Bayesian phylogenetic method implemented in BEAST v1.10.4^49^ was used to perform tip-dating analysis and estimation of the past population dynamics of the pathogen. The Bayesian time-aware GMRF Skyride coalescent tree prior^50^, and the random local relaxed clock model^51^ were applied. Tree topology search was constrained such that the two major clades of GBS ST283 identified are monophyletic. For the isolates of which only the sampling years were available, their tip dates were sampled from a uniform distribution of which the lower bound, mean, and upper bound were set to be equal to their sampling year, their sampling year plus 0.5, and their sampling year plus one, respectively. The best-fit nucleotide substitution model was determined to be TVMe under the Bayesian information criterion by ModelFinder^46^, and was used for the analysis. Posterior distributions were estimated using two independent Markov chain Monte Carlo (MCMC) samplings. Each chain was 500,000,000 steps long, and parameter values were logged every 50,000th step with the first 10% discarded as burn‐in. The two sampling chains were compared and combined. Effective sample sizes of all parameters were greater than 300, indicating that the two independent MCMC samplings gave similar posterior distributions of parameter values, and all parameters were sufficiently well sampled. Tracer 1.7.1^52^ was used to compute the median and 95% highest posterior density of the past effective population size.

### Ancestral state reconstruction

Reconstruction of ancestral states, including the past geographical locations and the presence of the *tet*(M) gene in the bacterial genome, was performed under the maximum likelihood framework implemented in PastML 1.9.33^53^. Ancestral states were inferred based on the states of the collected samples using the marginal posterior probabilities approximation (MPPA) method, and the F81 model was used in the analysis. The time-calibrated maximum clade credibility tree yielded from the tip-dating analysis was used as the best working phylogenetic hypothesis with the heights of the nodes being median node heights.

### Temporal signal detection

Temporal signal in the sequence data was assessed by using root-to-tip linear regression analysis. For a given tree, root-to-tip genetic distances were plotted against tip dates by using the *lm* function in R. The root placement of a tree was determined by minimizing the root-mean-squared error by using the *rtt* function, implemented in the R library *ape*. For the isolates of which only the sampling years were available, their sampling dates were set to their sampling year plus 0.5. Tip-date randomization was used to compute the null distribution (N = 1000) of the slope of the regression model (i.e. the temporal signal). The root placement of the tree with tip dates randomized was also re-estimated by using the function *rtt*, minimizing the root-mean-squared error. The temporal signal was considered significant if the p‐value was below 0.05.

## Supplementary Information

Supplementary Legends.

Supplementary Information 1.

Supplementary Table S1.

Supplementary Table S2.

Supplementary Table S3.

Supplementary Table S4.

## Data Availability

Sequence data generated by this study are openly available from the NCBI database; BioProject accession number: PRJNA792205; SRA accession numbers: SRR17330942–SRR17330953. Sequence of A5’s Tn*916* and its flanking sequences is also openly available from the NCBI GenBank database, accession number: OM049525.
