# Supplementary material for: Genomic epidemiology of Streptococcus agalactiae ST283 in Southeast Asia
Source: Sci Rep. 2022 Mar 9;12:4185. doi: 10.1038/s41598-022-08097-0 (PMC8907273; doi:10.1038/s41598-022-08097-0)
Supplement: Supplementary file 1 — Supplementary Legends. [file 41598_2022_8097_MOESM1_ESM.pdf]

Supplementary information

Supplementary Data S1: **Manually curated multiple sequence alignment of single nucleotide variants used to reconstruct the maximum likelihood phylogeny (Figure 1).**

Supplementary Table S1: Sequencing summary statistics and metadata of the 310 bacterial isolates analyzed in this study.

Supplementary Table S2: Genome mapping summary statistics. *Streptococcus agalactiae* ST283 SG_M1 (GenBank accession number: CP012419.2) was used as the reference genome in the read mapping.

Supplementary Table S3: Putative drug resistance genes in the bacterial genomes detected by ResFinder [25]**.** The significant thresholds for gene detection were 95% gene mapping coverage, 95% gene-wide nucleotide identity, and 0.5 gene mapping depth normalized by whole-genome mapping depth (computed across mapped regions only). To be considered as positive, contiguous full-length gene sequences must also be present in the drafted genome assemblies and could be detected by BLASTn.

Supplementary Table S4: Draft assembled genomes containing contiguous sequences exhibiting similarity to the reference Tn*916* element (GenBank accession number: U09422.1). The similarity detection was done by using BLASTn with default settings. Only BLAST hits with >500 bases are reported
